# The Effect of Dietary Lactic Acid Bacteria on Intestinal Microbiota and Immune Responses of Crucian Carp (*Carassius auratus*) Under Water Temperature Decrease

**DOI:** 10.3389/fmicb.2022.847167

**Published:** 2022-04-18

**Authors:** Yuan Liu, Haoxin Lv, Liping Xu, Kun Zhang, Yan Mei, Jun Chen, Min Wang, Yifei Guan, Huili Pang, Yanping Wang, Zhongfang Tan

**Affiliations:** ^1^Henan Key Laboratory of Ion-Beam Bioengineering, School of Physics and Microelectronics, Zhengzhou University, Zhengzhou, China; ^2^Henan Key Laboratory of Ion-Beam Bioengineering, School of Agricultural, Zhengzhou University, Zhengzhou, China; ^3^School of Food Science and Technology, Henan University of Technology, Zhengzhou, China; ^4^Xining Vegetable Technical Service Center, Xining, China

**Keywords:** crucian carp, *lactic acid bacteria*, intestinal microbiota, cytokines, water temperature decrease

## Abstract

Temperature changes have a great impact on fish feeding, intestinal microorganisms, metabolism, and immune function. Therefore, it is necessary to develop effective methods to enhance the survival rates and growth of fish under water temperature changes. *Lactic acid bacteria* (LAB) are promising immunostimulatory feed additive, as demonstrated by their beneficial effects in several fish species. This study investigated the short-term effects of dietary LAB on intestinal microbiota composition and immune responses of crucian carp (*Carassius auratus*) when water temperature decreased from 30 ± 1°C to 18 ± 1°C. *Lactococcus* (*L.*) *lactis* 1,209 and *L. lactis* 1,242 with potential probiotics isolated from the intestine of Qinghai naked carp (*Gymnocypris przewalskii*) were selected as feed additives for the crucian carp feeding experiment. A total of 225 commercially available healthy crucian carp (250 ± 10 g) of similar age were kept in 30°C water for a week and then immediately transferred to 18 ± 1°C water, assigned to three dietary treatments for a 16-day feeding trial randomly: (1) HC, diets without additives (the control group); (2) HT, diets with 10^6^ CFU/ml *L. lactis* 1,209; and (3) HL, with 10^6^ CFU/ml *L. lactis* 1,242. Each group was set up with 3 replicates and each with 25 fish. The results showed that the mortality rate of crucian carp in HC, HT, and HL group was 50, 27, and 33%, respectively. High-throughput sequencing results displayed that the composition of the intestinal microorganism varied dynamically in response to different treatments and water temperature decrease. Among them, compared with the HC group, a higher abundance of *Firmicutes* and *Proteobacteria*, and a lower of *Actinobacteria* appeared in HT and HL. The cytokines heat shock protein 70 (HSP-70) in crucian carp intestinal tract significantly decreased when water temperature decreased (*p* < 0.05).

## Introduction

In recent years, the rapid development of aquaculture has already surpassed the production of other terrestrial animals used for food such as cattle, poultry, and swine (FAO, 2020). Although the massive growth of aquaculture has improved the livelihood of farmers in many countries, the industry is seriously hampered by diseases which are the principal constraint of nurseries and grow-out mortalities in fish cultures (Loh et al., 2019). Intensive and inefficient, the outbreaks of bacterial diseases cause tremendous economic losses to the industry of aquaculture, especially bacterial infection diseases, including photobacteriosis, furunculosis, and vibriosis (Yukgehnaish et al., 2020). In order to prevent or control the infections, antibiotics have been widely used (Angela et al., 2020). However, antibiotic resistance is a huge concern in the aquaculture industry, and long-term use of these antibiotics could cause the survival of antibiotic-resistant bacteria which endangers the health of humans (Dawood et al., 2018). Therefore, it is necessary to develop alternative methods to enhance the survival rates and growth of cultured aquatic species without antibiotics (Shi et al., 2020b).

During past two decades, numerous studies revealed that modifications of gastrointestinal microbiota by antibiotics can alter the likely benefit of the host–microbiota interaction or relationship (Yukgehnaish et al., 2020). In addition, several studies have demonstrated that the intake of probiotics can modify the composition of the intestinal microbiota and effectively assist in returning the microbiota, which were disturbed by antibiotics or other risk factors, to its normal beneficial composition (Gomez and Balcazar, 2008). In order to address infectious bacterial diseases in the long term, the addition of probiotics, as a more suitable technique approach, is accepted by many scientists due to its necessity and how it provides less harm to the ecosystem (Yukgehnaish et al., 2020). Probiotics can effectively improve the growth rates and feeding efficiency of fish and regulate their gut microbiota (Iribarren et al., 2012; Bachruddin et al., 2018; Volkoff, 2019). Recent studies show that probiotics and prebiotics not only promote the growth performance but also fortify the immune system in cultured aquatic species. In aquaculture, among the available probiotics, *lactic acid bacteria* (LAB) have been widely studied for their probiotic properties (Kim et al., 2013; Ringo et al., 2018). Previous studies show that the proliferation of LAB in the intestine of fish suppresses the colonization of Gram-negative pathogenic bacteria (Kishawy et al., 2020). Moreover, aqua-feeds supplemented with LAB have been reported to stimulate the growth, enhance the disease resistance, effectively regulate the gut microbiota (Xia et al., 2020), and stimulate immune responses in cultured aquatic species (Ringo et al., 2018; Ashouri et al., 2020). LAB is expected to be growth promoters and immunostimulants in aquaculture (LeBlanc et al., 2017). It is well known that various factors, such as direct-fed microorganisms or diet composition, could alter the composition of intestinal microorganisms (Fan et al., 2021). Therefore, it is important for this study to investigate the effect of feed additives (with or without LAB) on intestinal microbiota. Consequently, the application of LAB blends in fish feed has been proposed as an optimal strategy to explore their potential positive effects on gut microbiota and immune responses on fish (Busti et al., 2020).

Crucian carp is an omnivore and one of the most important economic freshwater fish in China. It is abundant in rivers, lakes, and reservoirs throughout the country (Li et al., 2018; Luo et al., 2021). Crucian carp is chosen as a model fish for this study due to its palatability, high nutritional quality, and fast growth rate (Cui et al., 2020; Wu, 2020; Du et al., 2021). As is well known, environmental change can suppress fish immunity, leading fish to be infected by pathogens (Luo et al., 2021). In addition, environmental fluctuations that induce physiological stress were demonstrated to affect the survival, growth, physiological, and immunological functions of aquatic animals (Pai-Po et al., 2017). Emerging evidence have also shown that exposure to ambient stressors, such as temperature change, may trigger severe diseases (Qi et al., 2020). Therefore, temperature change may render fish to be less resistant against pathogenic infection.

Previous research has shown that acute changes in water temperature can affect body temperature of fish, induce changes their reaction rate of biological processes, and reduce their physiological performance (Yu et al., 2018). Hence, water temperature is a determinant environmental factor for species that are highly susceptible to temperature changes. Water temperature plays a crucial role in nutrient utilization, metabolism, and regulation of gut microbiota (Sánchez-Nuño et al., 2018a; Pelusio et al., 2021). Variations in temperature shape the composition of fish gut microbiota, particularly in lineages of Firmicutes and Proteobacteria (Mateus et al., 2017). Intestinal microbiota exerts a significant part in the regulation of host health. Increasing evidence has revealed that composition disturbances in fish gut microbiota are critical risk factors in disease development (Nie et al., 2019). To date, very limited studies have investigated the LAB diet effect on the gut microbiome structure in fish, especially in crucian carp, when the water temperature drops. Moreover, the precarious conditions of water temperature change disturb the gut microbiota of fish, which is not a choice for sustainable development of aquaculture.

There has been great interest to explore the relationship between the alteration of immune-related cytokine levels and the intestinal flora of crucian carp with LAB treatment under temperature change. Among immune parameters, cytokines have been commonly used as reference indicators in immunomodulatory studies (Liu et al., 2014; Mosca et al., 2014). In particular, several studies show that LAB and other probiotics can electively regulate the expression of proinflammatory and inflammatory cytokines including tumor necrosis factor (TNF)-α, interleukin (IL)-6, IL-1β, and IL-10, interferon (IFN)-γ, etc. (Liu et al., 2016; Zhou et al., 2018; Xia et al., 2019; Jang et al., 2020). On the other hand, immunomodulatory effects of LAB as feed additives have received increasing attention (Feng et al., 2016, 2019; Giri et al., 2016). In the present study, we aim to explore the effects of dietary LAB on the survival rate, intestinal microorganism, immune response and disease resistance of crucian carp against *Aeromonas hydrophila* ATCC 7966 (*A. hydrophila* ATCC 7966) under water temperature decrease. This research might contribute to help better understand the advantages involved in stimulating immune response and regulating gut microbiome of dietary LAB with water temperature change for crucian carp, which will be beneficial for understanding the positive influence of dietary LAB on aquaculture.

## Materials and Methods

### Experimental Design and Feeding Management

The experiment was carried out at the Laboratory of Aquaculture, Henan Key Laboratory of Ion-Beam Bioengineering, School of Agricultural Sciences, Zhengzhou University, Zhengzhou, China. Crucian carp were obtained from the vegetable markets around Zhengzhou University. The experiment was conducted in a recirculating system. At the beginning, the tanks were filled with water for 3 days and aerated continuously. Two hundred and thirty trial healthy fish were placed in 700 L (120*120*80 cm^3^) tanks at 30 ± 1°C for 1 week and fed with the basal diet. Then, 225 healthy crucian carps similar in length (18 ± 2 cm) and body weight (250 ± 10 g) were randomly distributed into three groups [the basal commercial diet, HC; a basal diet with 1 × 10^6^ CFU/ml of *Lactococcus lactis* 1,209 (*L. lactis* 1,209), HT; a basal diet with 1 × 10^6^ CFU/ml of *Lactococcus lactis* 1,242 (*L. lactis* 1,242), HL]. Twenty-five fish were randomly placed in each tank (70*50*40 cm^3^), and each experiment was conducted in triplicate. In preparing the experimental diets, the basic feed formula is shown in Table 1. The whole experiment lasted for 16 days. *L. lactis* 1,209 and *L. lactis* 1,242, obtained from Zhengzhou University, China, were selected by their strong immunomodulatory activities and adhesion capacities (Cui et al., 2018). Every tank was equipped with a filter and an oxygen supply device, and the water temperature was maintained at 18 ± 1°C throughout the experiment. The averages of pH and dissolved oxygen values were pH 8.0 and 6.3 mg/L, respectively. Fishes of all three groups were fed with common diets once daily at an amount that is 2% their body weight. The HT and HL groups were fed with the prepared feed once every 2 days. All animal experiments were performed in accordance with the legal and ethical regulations by Ethics Review Committee of Zhengzhou University (ZZUIRB2021-111).

**TABLE 1:** Composition of the basal commercial diet.

Nutrition facts	HC (%)	HT (%)	HL (%)
Protein	30	30	30
Crude fat	20	20	20
Crude fiber	4	4	4
Moisture	5	5	5
Algea	25	25	25
Other ingredients	18	18	18
*Lactococcus lactis* 1,209	0	10^6^ CFU/mL	0
*Lactococcus lactis* 1,242	0	0	10^6^ CFU/mL

*Ingredients: White fish meal, Fish protein, Flour, Soybean meal, Yeast, Antarctic shrimp meal, Natural immune protein, Organic minerals, Milk powder, Green algae, Red algae, etc.*

### Sample Collection

The fish survival conditions were recorded throughout the feeding experiment. During feed treatment, four fish were randomly selected from every group on 0, 5, 10, and 15 days. All the selected fish was anaesthetized with an overdose of ethyl 3-aminobenzoate methanesulfonate (MS-222, 200 mg L–1). The fishes’ body surfaces were wiped with 75% alcohol cotton. The total fish gut and the total intestine contents were aseptically harvested in a clean bench. Intestines were aseptically excised and intestinal contents were removed by using sterile scissors. Intestinal contents were obtained by gently squeezing the entire intestine. The total fish gut was aseptically removed, opened, and gently agitated three times in sterile phosphate buffered saline (PBS) to remove the contents and non-adhesive bacteria. These were then cut into small sections. Both gut tissues and intestinal contents were immediately frozen in liquid nitrogen and stored at −80°C for further analysis. The total fish gut tissues were collected for inflammation related cytokines, and the collected intestinal contents were used for microbial diversity analysis.

### High-Throughput Sequencing Analysis of Gut Microbiota

#### Genomic DNA Extraction

Deoxyribonucleic acid was extracted from fish intestinal contents samples using the bacterial DNA Kit (D3350-02, Omega Bio-tek, Norcross, GA, United States) according to the manufacturer’s protocol. Moreover, the quality and integrity of DNA were monitored using 1% agarose gel electrophoresis. The concentrations of DNA extracts were measured by a NanoDrop 2000 UV-Vis spectrophotometer (Thermo Scientific, Wilmington, DE, United States). The V3–V4 regions of bacterial 16S rDNA were selected for generating amplicons and subsequent taxonomy analysis with the primer set 338F (5’ -ACTCCTACGGGAGGCAGCAG-3’) and 806R (5’ -GGACTACHVGGGTWTCTAAT-3’) (Yang et al., 2019; Wang et al., 2020).

#### PCR Amplification

The PCR amplification procedure are as follow: pre-denaturation at 95°C for 3 min, followed by 29 cycles of 30 s at 95°C, 30 s annealing at 53°C, 45 s elongation at 72°C, and a final extension at 72°C for 10 min.

#### Illumina High-Throughput Sequencing of Barcoded 16S rRNA Genes

After purifying the PCR products, the samples were sequenced on the Illumina MiSeq platform (Shanghai Majorbio Bio-Pharm Technology Co., Ltd., China) to measure the diversity and bacterial composition in fish intestinal contents. Data of high throughput sequencing was analyzed on an online platform, the Majorbio Cloud Platform^1^.

Quality filtering and demultiplex on joined sequences was performed using the QIIME. After removing the low-quality sequences and reads, operational taxonomic units (OTUs) were generated by clustering with a 97% similarity threshold using UPARSE (version 7.0^2^). The microbial diversity in fish intestinal contents was estimated for the analysis of Venn diagram and alpha-diversities, which included the Shannon index, Simpson index, Chao1 index, and Ace index, using Mothur (version 1.30.2^3^). Beta-diversity was analyzed using PCA and Partial Least Squares Discriminant Analysis (PLS-DA) based on the Weighted-Unifrac distance matrix method. Furthermore, bar plots and heatmaps of species composition and difference analysis were created using R software (version 3.3.1), and a value of *p* < 0.05 was statistically significant.

### Immune-Related Cytokine Assays

The selected immune-related cytokines were TNF-α, IFN-γ, IL-10, IL-6, hsp70, and IL-1β. The gut tissue samples of crucian carp fed for 0, 5, 10, and 15 days, respectively, were selected. The levels of cytokines (as described above) in the gut were evaluated using ELISA kits (Beijing winter song Boyue Biotechnology Co., Ltd., China). The measurement was performed with commercial kits from Beijing winter song Boyue Biotechnology Co., Ltd. (Beijing, China) according to the manufacturer’s instructions. Briefly, the gut tissues were rinsed with ice-cold PBS (0.01 M, pH = 7.4) to thoroughly remove excess blood. Then, the samples were homogenized in PBS with a glass homogenizer on ice. The homogenates were centrifugated at 5,000 × *g* for 5 min to get the supernatant for further analysis. The immune-related cytokine assays were measured by using tetramethylbenzidine (TMB) as the substrate at 450 nm optical density (OD) with a Microplate Reader (Thermo Scientific). Finally, the concentration of immune-related cytokines in the samples was determined by comparing the OD value of the samples to the standard curve.

### Challenge Test With *Aeromonas hydrophila* ATCC 7,966

After the sample collection, crucian carp in all groups (HC, HT, and HL) were fed as before. Fishes in the HC groups were divided into two groups, HC1 group (the basal commercial diet) and *A. hydrophila* group (a basal diet with 1 × 10^5^ CFU/ml of *A. hydrophila* ATCC 7966). Every experiment group was conducted with 8 healthy fish. The survival was monitored in all groups after being fed with *A. hydrophila* ATCC 7,966, *L. lactis* 1,209, and *L. lactis* 1,242, respectively. Strain *A. hydrophila* ATCC 7,966 was grown at 37°C for 12 h in nutrient agar (NA), and the feed diet was prepared with the same method as before. The concentration of *A. hydrophila* ATCC 7,966 was 10^5^ CFU/ml of feed diet. The fish were fed once daily. The fish in all groups were observed daily, and dead fish were immediately removed from the tanks. Mortality rates were also recorded daily for each group for 12 days after challenge test.

### Statistical Analysis

Microbial communities and immune-related cytokines were analyzed using IBM SPSS Statistics 25.0 and Origin 2017. Two-way ANOVA procedures were used to compare the means with a significant difference of *p* < 0.05. Duncan’s multiple range method comparisons were used to compare differences among all groups.

## Results

### Mortality Rate

The number of the dead carp was recorded, and the mortality rate was calculated during the process of the experiment. As shown in Table 2, the mortality rate of HC (50%) was significantly higher than that of HT (27%) and HL (33%). The results showed that the mortality of crucian carp in the LAB treatment group was lower than in the control group under the condition of temperature change.

**TABLE 2:** Mortality and sequence number based on operational taxonomic unit (OTU) levels in crucian carp.

Group	Mortality(%)	Sequence number	OTU number	Coverage
HC	50	56,516	1,345	0.99733
HT	27	55,590	1,561	0.99760
HL	33	54,558	1,509	0.99738

*HC: The control group, HT: The L. lactis 1,209 treatment group, HL: The L. lactis 1,242 treatment group.*

### Intestinal Microbiota Analyses

A total of 3,313,921 effective sequences were obtained through 16S rRNA high-throughput sequencing of the V3–V4 region in the present study, with an average sequence length of 416 bp. As shown in Table 2, the mean valid sequences from HC, HT, and HL samples were 56,516, 55,590, and 54,558, respectively. These reads were clustered into a total of 2,478 OTUs based on 97% sequence identity. The average Good’s coverage for samples was higher than 99%, indicating that the majority of the microbial species present were identified and sequencing depth was also adequate for robust sequence analysis.

### Richness and Diversity

To compare and estimate the bacterial diversity in every group when the temperature suddenly changed, intestinal microbiota community diversity and richness indices were calculated from the proportion of OTUs. As shown in Table 3, on the 5th day, the species diversity (Shannon index) in HT and HL groups was higher than that of the HC groups, and all groups were lower than that of HC group at the 0 day. However, the Simpson index showed otherwise, which signified that the diversity for the all groups decreased, and the HT and HL groups were higher than the HC group. The species richness Chao and Ace indices in HT and HL groups at 5 days was higher than that of the HC group, and all groups at 5 days were lower than that of the HC groups at 0 day, which signified the abundance for the LAB treatment groups was higher than the HC group under the sudden decrease of water temperature.

**TABLE 3:** Alpha diversity indices of intestinal microbiota based on OTU levels in every group of crucian carp under water temperature decrease.

		Coverage	Diversity index		Richness estimato	
			Shannon	Simpson	Ace	Chao
HC	0 day	0.99772 ± 0.001	4.0799 ± 0.567^a^	0.05591 ± 0.035^ab^	487.28 ± 28.42^ab^	485.4 ± 23.457^ab^
	5 day	0.99867 ± 0.0004	1.7332 ± 0.513^ab^	0.4005 ± 0.194^ab^	215.32 ± 79.72^a^	187.31 ± 31.063^a^
	10 day	0.99675 ± 0.003	3.5256 ± 0.387^ab^	0.10256 ± 0.050^ab^	521.05 ± 256.52^ab^	523.66 ± 254.59^ab^
	15 day	0.99672 ± 0.0007	2.9509 ± 1.00^ab^	0.21192 ± 0.174^ab^	506.34 ± 84.69^b^	497.4 ± 92.986^b^
HT	5 day	0.99815 ± 0.001	1.9385 ± 1.15^ab^	0.37588 ± 0.212^ab^	306.01 ± 202.03^ab^	292.77 ± 207.92^ab^
	10 day	0.99804 ± 0.0009	1.6681 ± 0.504^ab^	0.48613 ± 0.087^a^	297.85 ± 144.28^ab^	292.81 ± 150.69^ab^
	15 day	0.99701 ± 0.002	2.7821 ± 0.823^ab^	0.26546 ± 0.150^b^	465.3 ± 265.28^b^	468.13 ± 263.44^b^
HL	5 day	0.99847 ± 0.0006	2.7448 ± 1.39^ab^	0.24844 ± 0.226^ab^	332.98 ± 173.9^ab^	317.2 ± 190.03^ab^
	10 day	0.99685 ± 0.002	1.8005 ± 1.13^a^	0.49453 ± 0.327^ab^	466.83 ± 329.06^ab^	404.98 ± 248.34^ab^
	15 day	0.99681 ± 0.001	3.8 ± 0.669^b^	0.12125 ± 0.1^ab^	602.77 ± 136.25^b^	611.47 ± 139.73^b^

*Values were expressed as means ± S.E (n = 3), and values within the same row with different letters are significantly different (p < 0.05). HC: The control group, HT: The L. lactis 1,209 treatment group, HL: The L. lactis 1,242 treatment group.*

The effects of LAB diet on intestinal microbiota community diversity and richness in crucian carp was evaluated based on alpha diversity, as shown in Table 3. In general, the Shannon, Chao, and Ace indices of the three groups decreased first and then increased with time, and the HT and HL groups were higher than that of HC group throughout the period. However, the Simpson index showed the opposite trend. The Shannon index of HL group on the 15th day was significantly higher than on the 10th day (*p* < 0.05). On the other hand, Simpson index was significantly higher between the 10th and 15th day in the HT group (*p* < 0.05). Chao and Ace index of HC group on the 5th day were significantly lower than that on the 15th day (*p* > 0.05), and the diversity value on 15th day of the HL group was the highest.

### Bacterial Composition Analysis of Intestinal Microbiota

**FIGURE 1:**
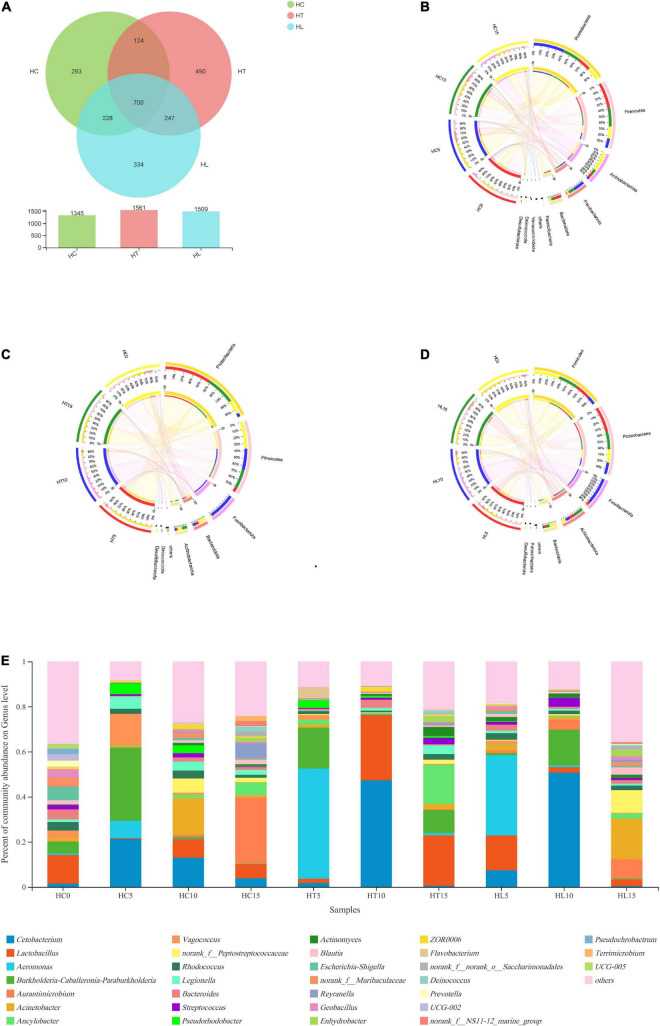
Beta diversities of intestinal bacterial community composition of crucian carp (*Carassius auratus*). **(A)** A Venn diagram showing bacterial numbers shared within and between sample groups. A circos plot at the phylum levels shows the linkage among **(B)** the control group (HC), **(C)** the *L. lactis* 1,209 treatment group (HT), and **(D)** the *L. lactis* 1,242 treatment group (HL), with 10^6^ CFU/mL *L. lactis* 1,242. **(E)** Bacterial community and relative abundance at the genus levels for all crucians. Taxa with an abundance < 2% are included in “others.” HC: The control group, HT: The *L. lactis* 1,209 treatment group, HL: The *L. lactis* 1,242 treatment group.

The effects of LAB as feed additive on species composition were shown in Figures 1A–E. The Venn diagram in Figure 1A presents the bacterial communities’ unique or common OTUs between any two treatments. The number of OTUs detected between the HC, HT, and HL groups was 1,345, 1,561, and 1,509, respectively, of which 700 were common OTUs to all groups, accounting for 28.25% of total observed OTUs (2478). Moreover, 124, 228, and 247 OTUs were shared by the HC group and HT samples, the HC group and HL samples, and HT samples and HL samples, respectively.

Based on Illumina platform analyses, microbial composition and relationships at the phylum level were shown (Figures 1B–D). The top five predominant bacterial phyla in the intestine of the crucian carp contained *Proteobacteria, Firmicutes, Actinobacteria, Bacteroidetes*, and *Fusobacteria*. Additionally, compared with the HC group, *Firmicutes* and *Proteobacteria* increased in the HT and HL groups. On the other hand, *Actinobacteria* decreased from 20 (HC) to 6.9% (HT) and 13% (HL), respectively.

Taxon-dependent analysis was used to compare the relative abundance of bacterial genus in the intestinal contents of crucian carp fed with different contents of LAB diets with the sudden decrease of temperature (Figure 1E). At the genus level, the top 8 most dominant genera of the crucian carp intestinal microbiota communities were the *Cetobacteria*, *Lactobacillus, Aeromonas, Burkholderia, Aurantimicrobium, Acinetobacter, Ancylobacter*, and *Vagococcus*. On the one hand, the relative abundance of *Aeromonas* and *Lactobacillus* significantly increased in the HT and HL groups compared to those in the HC group at 5 days (*p* < 0.05). In addition, the abundance of *Cetobacteria* and *Burkholderia* significantly decreased in the HT and HL groups compared with the HC group (*p* < 0.05). These results indicate that the decrease of the temperature disrupted the homeostasis of intestinal microbial composition, causing a stress response in crucian carp. On the other hand, with the extension of culture, the abundance of intestinal microbiota changed greatly, including the abundances of *Cetobacteria, Lactobacillus, Aeromonas*, and *Burkholderia*. On the 10th day, the abundance of *Cetobacteria* was significantly higher than that of the HT and HL groups compared with the HC group (*p* < 0.05). On the 15th day, the abundance of *Lactobacillus* was decreased in the HL group and increased in the HT group compared with that of the HC group. In general, the relative abundance of the samples with three different treatments significantly changed with the obvious change of environmental temperature at the genus level.

### The Principal Component Analysis (PCA) and Partial Least Squares Discriminant Analysis (PLS-DA)

**FIGURE 2:**
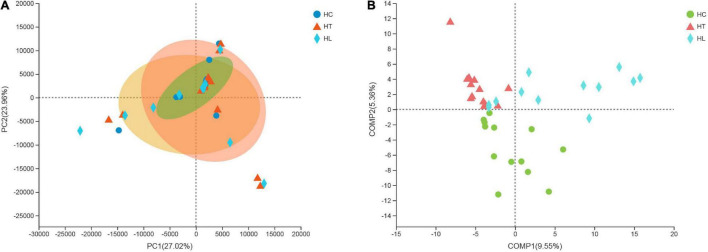
Principal coordinate analysis (PCoA) plot **(A)** and the supervised discriminant analysis with Partial Least Squares Discriminant Analysis (PLS-DA) plot **(B)** based on QIIME software between intestinal microbiota samples from HC, HT, and HL groups. HC: The control group, HT: The *L. lactis* 1,209 treatment group, HL: The *L. lactis* 1,242 treatment group.

According to beta diversity analyses, the overall structural changes at the genus level of the gut microbiota were analyzed using unsupervised multivariate statistical methods of the Principal Component Analysis (PCA) with QIIME software. As shown in Figure 2A, the area of the group ellipse of sample points in the three groups of different treatments was significantly different, and the area of group ellipse in the HT and HL groups was significantly larger than that in HC group. PC1 axis and PC2 axis were 27.02 and 23.96%, respectively. Moreover, in order to make the differences among three groups more obvious, the supervised discriminant analysis of PLS-DA was further used to analyze the intestinal contents samples of crucian carp (Figure 2B). Samples from fish in the HC, HT, and HL groups were clearly scattered in different quadrants. The results indicated that the microbial composition of the samples of crucian carp intestinal content was significantly different from the HC, HT, and HL groups when the temperature decreased.

### Analysis of Species Differences

**FIGURE 3:**
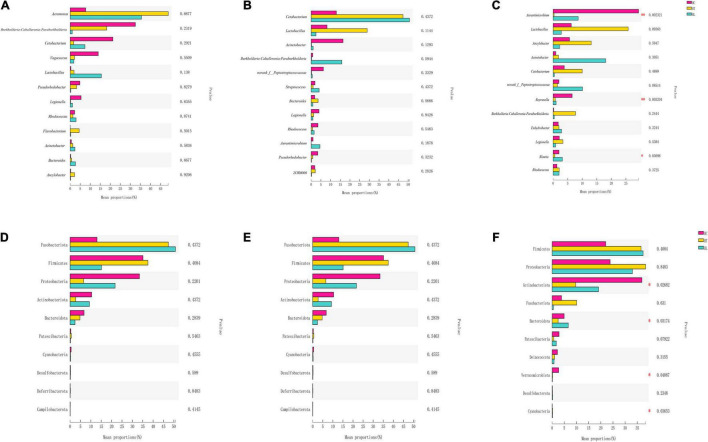
Significant differences in relative abundance of the top 12 genera and top 10 phylum in different periods. **(A)** 5 days at the genera level, **(B)** 10 days at the genera level, **(C)** 15 days at the genera level, **(D)** 5 days at the phylum level, **(E)** 10 days at the phylum level, and **(F)** 15 days at the phylum level. ANOVA was chosen to find the differential abundance using a significance threshold of *p* < 0.05. “*”indicates *p* < 0.05; “^**^” indicates *p* < 0.01. HC: The control group, HT: The *L. lactis* 1,209 treatment group, HL: The *L. lactis* 1,242 treatment group.

**FIGURE 4:**
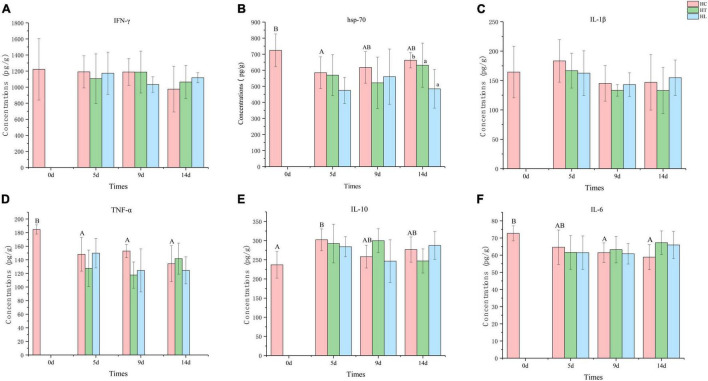
The concentration of different immune-related cytokines in different intestinal samples. **(A)** IFN-γ, **(B)** HSP-70, **(C)** IL-1β, **(D)** TNF-α, **(E)** IL-10, and **(F)** IL-6. Values are given as the mean ± SD (*n* = 10–12). HC: The control group, HT: The *L. lactis* 1,209 treatment group, HL: The *L. lactis* 1,242 treatment group.

The Kruskal-Wallis *H* test was performed to evaluate the differences among different groups (Figure 3). According to different days, species differences were analyzed in 12 genera in HC, HT, and HL groups. As shown in Figures 3A–C, the species composition at 5 (Figure 3A) and 10 days (Figure 3B) showed no significant differences. At the 15th day (Figure 3C), however, there was extremely significant difference between *Aurantimicrobium* and *Reyranella* (*p* ≤ 0.01). *Blautia* also showed significant differences (*p* ≤ 0.05). In addition, species differences were analyzed in 10 phyla among intestinal contents samples in HC, HT, and HL groups Figures 3D–F. The results were similar with that of genus level, while *Actinobacteriota, Bacteroidota, Verrucomicrobiota, Cyanobacteria* showed significant differences (*p* ≤ 0.05) at the 15 day (Figure 4F).

### Assay of Immune-Related Cytokines Using ELISA

The levels of pro-inflammatory cytokines TNF-α, IL-6, IL-1β, anti-inflammatory cytokines IL-10, and IFN-γ and synergistic immune activity of HSP-70 were examined. As shown in Figure 4, with the temperature suddenly decreasing, the concentration of HSP-70, TNF-α, and IL-6 in all groups were lower at 5 days than that of 0 day, especially in the HT and HL groups which were lower than of the HC group. The concentration of IL-1β and IL-10 of all groups were higher at 5 day than at 0 day, and IL-10 in the HC group was significantly higher (*p* ≤ 0.05) between 5 days and 0 day. The concentration of IFN-γ (Figure 4A) decreased with time, and the HT and HL groups were higher than that of HC group on the 15th day. From the results of HSP-70 (Figure 4B), it can be seen that the HT and HL groups were always lower than that of HC group, and the concentration of HT and HL groups on the 15th day were significantly lower than that of the HC group (*p* ≤ 0.05). In general, IL-1β (Figure 4C) did not change significantly over time, and concentrations were highest at 5 days for all three groups. The concentration of TNF-α (Figure 4D) decreased with time, the HT and HL groups were higher than that of HC group at 15 day, and the highest concentration was found in the HT group. Overall, the concentration of IL-10 (Figure 4E) increased with time, and the highest concentration was found in the HT group at 15 days, whereas HT group was highest on 10 days. The concentration of IL-6 (Figure 4F) in the HC group was decreased, and the concentration of IL-6 in the HT and HL groups were higher than the HC group at 15 days.

### The Relationship Between Immune-Related Cytokines and Gut Bacterial Communities

**FIGURE 5:**
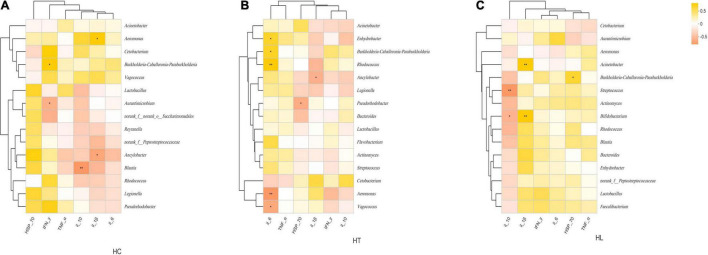
Spearman’s correlation heatmaps of the top 15 genera and immune-related cytokines at **(A)** HC, **(B)** HT and **(C)** HL groups. The *X* and *Y* axes are environmental factors and genus, respectively. Yellow indicates a positive correlation, whereas red indicates a negative correlation. “*” indicates *p* < 0.05; “**” indicates *p* < 0.01. HC: The control group, HT: The *L. lactis* 1,209 treatment group, HL: The *L. lactis 1,242* treatment group.

Correlation heatmap showed that the relationship between immune-related cytokines and gut bacterial communities at the genus level in different intestinal samples varied (Figure 5). In the HC group (Figure 5A), IFN-Y was significantly and positively related to *Burknerella*, but significantly (*p* < 0.05) and negatively related to *Aurantimicrobium*. IL-10 was significantly and negatively related to *Blautia* (*p* < 0.01). IL-1β was significantly and positively correlated to *Aeromonas*, and significantly and negatively correlated to *Ancylobacter* (*p* < 0.05). In the HT group (Figure 5B), IL-6 was significantly (*p* < 0.05) and positively correlated to *Burkholderia, Rhodococcus* (*p* < 0.01), and *Enhydrobacter*, and significantly (*p* < 0.05) and negatively correlated with *Aeromonas* (*p* < 0.01) and *Vagococcus*. HSP-70 was significantly and negatively correlated with *Pseudorhodobacter* (*p* < 0.05). IL-1β was significantly and negatively related to *Ancylobacter* (*p* < 0.05). In the HL group (Figure 5C), IL-10 was significantly and negatively correlated with *Bifidobacterium* (*p* < 0.05) and *Streptococcus* (*p* < 0.01). IL-1β was significantly (*p* < 0.01) and positively related to *Acinetobacter* and *Bifidobacterium*. HSP-70 was significantly (*p* < 0.05) and positively correlated to *Burkholderia*.

### Disease Resistance

**FIGURE 6:**
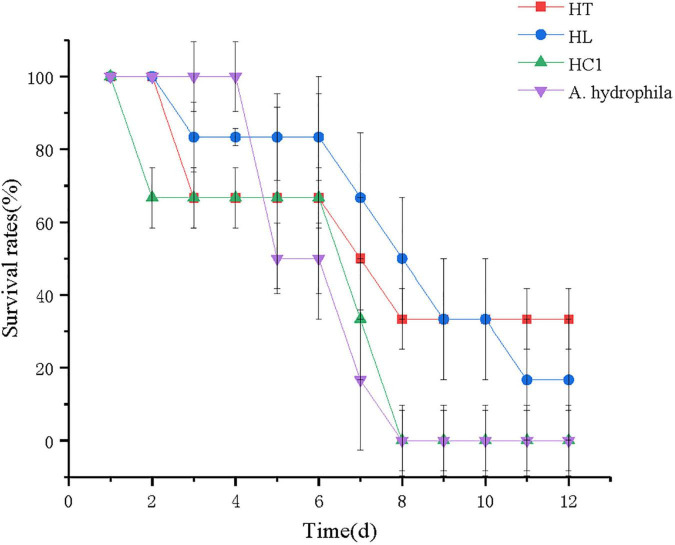
Survival rates of crucian carp (*Carassius auratus*) fed with experimental diets. HC: The control group, HT: The *L. lactis* 1,209 treatment group, HL: The *L. lactis* 1,242 treatment group, *A. hydrophila*: The *A. hydrophila* ATCC 7,966 treatment group.

The survival rate of crucian carp after the 12-day challenge experiment is shown in Figure 6. The survival rate of fish fed with *L. lactis* 1,209 were significantly higher than that of HC1 group. At 12 days, the survival rate of the crucian carp in both *A. hydrophila* group and the HC1 group was 0%, which was lower than that of the HT (30%) and HL group (20%). Additionally, the crucian carp in the HC1 group and the *A. hydrophila* group died relatively earlier than that of the HT and HL groups.

## Discussion

The intestinal microflora is a complicated and giant ecosystem that has a mutualistic relationship with its human or animal host. The system plays an important role in being a barrier against harmful microorganisms and various toxins (Xia et al., 2019). The immune system is critical to maintaining the health of the body as it is a defense against pathogenic microorganisms (Wolf and Underhill, 2018; Meng et al., 2019). In this study, the changes of intestinal microflora, immune-related cytokine expression with decreased water temperature were investigated to determine the short-term effects of the additive LAB on the microflora and immunity of crucian carp flora.

After 16 days of feeding trial, the data presented in this study indicated that LAB diet had a positive effect on the mortality rate of crucian carp compared to that of HC group when the water temperature decreased. In fact, “winter mortalities” phenomenon has been reported at persistent low temperature during the winter. The gradual reduction of water temperature often causes growth arrest and metabolic depression for a large number of fish species (Sánchez-Nuño et al., 2018a). Similarly, Sanchez-Nuno et al. also reported the “winter growth arrest,” where gilthead sea bream showed a doubling of feed conversion ratio (FCR) and a fourfold drop of specific growth rate (SGR) from 22°C to 14°C (Sánchez-Nuño et al., 2018b). Accumulating evidence additionally indicated that low temperature contributed to susceptibility to diseases in cultured species (Ibarz et al., 2010). In particular, previous studies have suggested that variations in temperature (also includes low temperature) shape the composition of the gut microbiota across the fish (Sánchez-Nuño et al., 2018a), and the gut microbiota gradually stabilizes as fish adapt to their environment.

The intestinal microflora composition affects the host health and is diet-dependent. Therefore, assessing what impact feeding fish with LAB diets has on fish gut microbiota is essential to fully evaluate current aquaculture feeding strategies (Cláudia et al., 2021). Furthermore, considerable evidence shows that probiotics from the same autochthonous source have a great chance of colonizing the host gut, thereby bringing health benefits to the host (Sun et al., 2010; Mohammadian et al., 2016). In the present study, *L. lactis* 1,209 and *L. lactis* 1,242 were selected from the intestinal contents of Qinghai naked carp whose probiotic properties, including antibiotic sensitivity, gastrointestinal survival rate, bacteriostatic activity, bile salt tolerance, temperature range, high salt tolerance and so on, had been previously studied (Cui et al., 2018). The intestinal microflora affects physiological processes, immunological stress, and preventive infections in several hosts (Butt and Volkoff, 2019; Shi et al., 2020a). In aquaculture, an increasing number of studies have focused on probiotics (Akhter et al., 2015), among which LAB was most widely used (Kim et al., 2013; Ringo et al., 2018). LAB has been proven to be effective in modifying the host-associated intestinal microbiota (Xia et al., 2018; Ringo et al., 2020). The intestinal microflora play an important role in fish, such as participating in a variety of physiological functions including feed digestion, reduction of the invasion of pathogenic bacteria, and synthesis of the trace elements, amino acids, and vitamins (Mente et al., 2016; Gao et al., 2019; Siddik et al., 2020). It is well known that various factors could alter the composition of intestinal microorganisms, such as direct-fed microorganisms or diet composition (Fan et al., 2021). Therefore, it was important for this study to investigate the effect of the feed additives (with or without LAB) on intestinal microbiota. The present study employed high-throughput sequencing to investigate the intestinal microbiota of crucian carp fed with LAB. The results showed that decreased microbial community richness, diversity after feeding LAB diet under the water temperature decrease, and the abundance for the LAB treatment groups was higher than that of the control group. In aquaculture, probiotic supplementation is known to alter the host’s gut microbial diversity (Wang et al., 2017; Niu et al., 2018). In accordance with our data, Jang et al. (2020) showed microbial diversity reduced compared to the initial group, and the probiotics treatment group had higher richness and diversity estimates than the control group of juvenile olive flounders (*Paralichthys olivaceus*).

According to previous reports, regardless of the diet, the gut microbiota in vertebrates is enriched with the phyla *Firmicutes, Actinobacteria, Proteobacteria, Bacteroidetes*, and *Fusobacteria* (Sepulveda and Moeller, 2020), consistent with our results. Furthermore, we observed a higher abundance of *Firmicutes* and *Proteobacteria*, and a lower abundance of *Actinobacteria* in the LAB treatment groups when compared with the control group. These results agreed with a recent study showing relative abundances of *Firmicutes* and rearing temperature have negative associations in rainbow trout (*Oncorhynchus mykiss*) (Huyben et al., 2018). Moreover, a previous study has shown that many of the compositional changes in the fish gut microbiota in response to temperature change that have been observed are driven by shifts in the relative abundances of *Proteobacteria* lineages in all fish species (Sepulveda and Moeller, 2020). For instance, the intestinal microflora of yellowtail kingfish (*Seriola lalandi*) found that increasing temperatures were associated with shifts in the relative abundances of *Gammaproteobacteria* lineages (Soriano et al., 2018). Similar shifts in Gammaproteobacteria abundances have been observed in salmon (*Salmo salar*). In addition, it was found that increasing temperatures were associated with decreases in the relative abundances of *Acinetobacter*, consistent with our results (Neuman et al., 2016). Additionally, the gram-positive bacteria phyla *Actinobacteria* in mice decreases after probiotic feeding with a high-fat diet (Azad et al., 2018; Liang et al., 2019). It is evident that LAB diet plays an important role in maintaining the intestinal microflora ecosystem in crucian carp under the water temperature decrease.

Furthermore, at the genus level, *Cetobacteria*, *Lactobacillus*, and *Aeromonas* increased in the HT and HL groups. In particular, the *Lactobacillus* in HT group increased the most, and compared with the HC group, *Acinetobacter*, *Burkholderia*, and *Aurantimicrobium* decreased in the HT and HL groups. According to previous reports, the gut microbiota in freshwater fish species is enriched with members of the family enterobacteriaceae representatives, including of the genera *Pseudomonas*, *Flavobacterium*, *Aeromonas*, *Acinetobacter*, and obligate anaerobic bacteria of the genera *Fusobacterium, Clostridium*, and *Bacteroides* (Gomez and Balcazar, 2008). Moreover, various species of LAB have also been proven to comprise part of this microbiota (Ringo and Gatesoupe, 1998; Balcazar et al., 2007), which is consistent with our results. Our study agrees with what was described by Shi et al. (2020b) over the 12-week trial period. Although the microbial diversity decreased of all treatments, the microbial community structure converged among treatments. Accordingly, these data suggested that the crucian carp feed with or without LAB affected the proportion of bacteria in the intestine, and the dominant bacteria in the LAB treated group tended to be evenly distributed and tended toward healthy levels. In conclusion, LAB for the feed additives can change the intestinal microbiota of crucian carp by regulating the balance of intestinal microbial ecosystem structure under the condition of temperature change.

In the fish immune system, cytokines play an important role. In particular, several studies showed that LAB and other probiotics can electively regulate the expression of proinflammatory and inflammatory cytokines including tumor necrosis factor (TNF)-α, interleukin IL-6, IL-1β, IL-10, and interferon (IFN)-γ which are commonly used as reference genes in immunomodulatory studies (Galindo-Villegas et al., 2012; Ashraf et al., 2014; Angela et al., 2020). On the other hand, immunomodulatory effects of LAB as a feed additive have recently received increasing attention as potential selection parameters for probiotics (Feng et al., 2016, 2019; Giri et al., 2016). In the present study, dietary LAB showed anti-inflammatory effects on the intestine of fish *via* decreasing the concentration of pro-inflammatory cytokines TNF-α and IL-1β, especially in the HT group. Our results showed that dietary LAB could decrease the concentration of TNF-α and IL-1β, which were consistent with that of Ou et al. (2019). It was reported that TNF-α and IL-1β enhance the inflammatory response by promoting the recruitment and activation of other inflammatory factors, thereby increasing the production and release of inflammatory mediators (Sanchez-Munoz et al., 2008). In this study, IL-6 was increased in the HT and HL groups. These differences of proinflammatory cytokines can be influenced by intestinal microbiota. It is well known that anti-inflammatory cytokines play an important role in maintaining immune homeostasis and preventing damage to host tissues by limiting pathogen activity (Iyer and Cheng, 2012). Interleukin IL-10 is a representative anti-inflammatory cytokine and is generally studied as a cytokine to identify immunoregulatory mechanisms (Jang et al., 2020). IL-10 was increased only in the HL group, which indicated that this group had a better immune balance. The results were in agreement with the previous studies on juvenile olive flounder *(Paralichthys olivaceus*) (Jang et al., 2020). INF- γ is one of the most important interferons in fish immunity (Zou and Secombes, 2011; Shukry et al., 2021). Heat shock protein 70 (HSP-70) is a stress marker in aquatic animals (Lindquist and Craig, 1988; Iwama et al., 1999; Shukry et al., 2021). In our study, there was no significant difference in INF-γ concentration among the groups, and HSP-70 was decreased in the HT and HL groups compared with the control group. In a similar sense, the mRNA expression levels of HSP-70 were increased of the Nile tilapia when exposed to toxicants and stressors (Abdel-Latif et al., 2021a,b). Additionally, previous research revealed that probiotics *Clostridium butyricum* could similarly increase the expression of the IL-10 and HSP-70 in weaning rex rabbits (Liu et al., 2019) and colon cancer HT-29 cells (Andriamihaja et al., 2009; Meng et al., 2021). In conclusion, these results suggest that the addition of LAB to the diet can stimulate the intestinal immune reaction of crucian carp. However, in aquaculture, the effects of dietary supplementation of probiotics on cytokines have been extensively studied, but results and expression levels have been inconsistent among many reports. For example, dietary *L. casei* BL23 could significantly increase the survival rate of zebrafish against *A. hydrophila* by enhancing the levels of anti-inflammatory cytokine IL-10 and pro-inflammatory cytokines IL-1β and TNF-α at mRNA levels (Qin et al., 2018). Qin et al. (2014) demonstrated that the dietary oligochitosan could significantly improve the growth performance and enhance in resistance against *A. hydrophila* of tilapia (*Oreochromis niloticus*) by reducing the levels of mRNAs encoding the stress-response HSP-70 and the pro-inflammatory protein TNF-α, while the diets increased the pro-inflammatory protein TNF-β (Shi et al., 2020a). Moreover, Shi et al. (2020b) showed that grass carp treated with the probiotic *B. subtilis* H2 revealed a significantly improved immune response in terms of upregulated mRNA levels of inflammatory cytokines IL-11, TNF-α, and IL-8. These inconsistent results could be manipulated by a number of factors, such as the water environment factors, the health status of animals, and the usage mode, dosage, and time of applying the probiotics. Hence, further research needs to be done to investigate the mechanisms by which probiotics are added to regulate animal health.

The activity and composition of intestinal microbiota influences intestinal environments, and therefore cytokines profiles in intestinal content (Liu et al., 2013) which exert vital influence on intestinal health. These cytokines may contribute to the regulation immune response and maintenance of homeostasis of the immune system, enhancement of host defense mechanism against bacteria and fungi (Siddik et al., 2020), lysozyme synthesis, and bactericidal activities (Giri et al., 2015). For example, Jang et al. (2020) reported that probiotics inspired pro-inflammatory responses by promoting proinflammatory (TNF-α, IL-6, and IL-1β) and anti-inflammatory cytokine (IL-10) production. Additionally, probiotic supplementation changed the expression levels of proinflammatory (Hasan et al., 2018) and anti-inflammatory cytokines (Iyer and Cheng, 2012) in fish in previous studies. In our study, due to changes in cytokine levels and bacterial abundance at the genus level, correlations between bacterial abundance and cytokines in different intestinal conditions varied.

## Conclusion

The probiotics, *L. lactis* 1,209 and *L. lactis* 1,242, showed the ability to adjust the composition of intestinal microbiota, stimulate secretes cytokines, and improve the survival rate and disease resistance against *A. hydrophila* in the crucian carp. In the present study, high-throughput analysis was used to investigate the dynamic changes of intestinal bacterial diversity under water temperature change and demonstrate beneficial effects of dietary LAB in gut compartments of crucian carp. Due to the limited information of LAB usage in crucian carp, whether dietary LAB alters the balance of intestinal microbes under water temperature change and affect host mortality and the mechanisms of immune response remains unknown. Therefore, further investigations are needed to reveal the detailed mechanisms of growth promotion and immune enhancement in crucian carp under water temperature change.

## Data Availability Statement

The datasets presented in this study can be found in online repositories. The names of the repository/repositories and accession number(s) can be found below: https://www.ncbi.nlm.nih.gov/, PRJNA788362.

## Ethics Statement

The animal study was reviewed and approved by the Institutional Animal Care and Use Committee at Zhengzhou University, Zhengzhou, China.

## Author Contributions

ZT designed the experiments. LX performed the investigation. YL carried out the experiments. YL, KZ, YM, MW, and YG analyzed the experimental results. YW, HP, and ZT interpreted the data. YL and HL wrote and edited the manuscript. All authors have read and agreed to the published version of the manuscript.

## Conflict of Interest

The authors declare that the research was conducted in the absence of any commercial or financial relationships that could be construed as a potential conflict of interest.

## Publisher’s Note

All claims expressed in this article are solely those of the authors and do not necessarily represent those of their affiliated organizations, or those of the publisher, the editors and the reviewers. Any product that may be evaluated in this article, or claim that may be made by its manufacturer, is not guaranteed or endorsed by the publisher.

## Footnotes

1
http://www.majorbio.com

2
http://www.drive5.com/uparse/

3
https://www.mothur.org/wiki/Download_mothur
