# Erratum: The effect of dietary lactic acid bacteria on intestinal microbiota and immune responses of crucian carp (*Carassius auratus*) under water temperature decrease

**DOI:** 10.3389/fmicb.2022.976726

**Published:** 2022-07-12

**Authors:** Frontiers Production Office

**Affiliations:** Frontiers Media SA, Lausanne, Switzerland

**Keywords:** crucian carp, *lactic acid bacteria*, intestinal microbiota, cytokines, water temperature decrease

Due to an Editorial error, the article was published in the incorrect journal section. This article's specialty section has been corrected to Microbial Symbioses, a section of the journal *Frontiers in Microbiology*.

The publisher apologizes for this mistake. The original version of this article has been updated.

